# Household food insecurity, coping strategies, and nutritional status of pregnant women in rural areas of Northern Ghana

**DOI:** 10.1002/fsn3.506

**Published:** 2017-08-27

**Authors:** Mahama Saaka, Jeremiah Oladele, Asamoah Larbi, Irmgard Hoeschle‐Zeledon

**Affiliations:** ^1^ School of Allied Health Sciences University for Development Studies Tamale Ghana; ^2^ International Institute of Tropical Agriculture Tamale Ghana; ^3^ International Institute of Tropical Agriculture Ibadan Nigeria

**Keywords:** food coping strategies, food insecurity, maternal nutrition, Northern Ghana

## Abstract

There is limited information on the magnitude and determinants of household food insecurity (HFI) and how it relates to the nutritional status of pregnant women in Northern Ghana. The magnitude, determinants of HFI, and how it relates to the nutritional status of pregnant women were evaluated in the Africa RISING West Africa project intervention communities in Northern Ghana. The prevalence of moderate and severe household hunger was 25.9% (95% CI: 19.0, 34.3) and 6.8% (95% CI: 4.2, 10.9) respectively. The independent predictors of maternal thinness were region of residence, gestational age and maternal age. Compared to women in the first trimester, women in the third trimester were 2.2 times more likely of being underweight adjusted odds ratio (AOR = 2.19, CI: 1.02, 4.70). Women who were under 20 years of age were 11.9 times more likely of being thin compared to women aged more than 35 years (AOR = 11.97, CI: 2.55, 5. 67). Food insecurity was highly prevalent but it was not associated with maternal thinness of pregnant women. The risk of maternal thinness increased as the gestational age increased and this has a great potential of adversely influencing pregnancy outcomes and overall quality of life.

## 1 INTRODUCTION

It is well documented that adequate nutritional status of women especially during pregnancy is crucial for child survival because an undernourished mother is more likely to deliver an infant with low birth weight, significantly increasing its risk of dying (Abu‐Saad & Fraser, 2010; Black et al., 2008; El‐Farrash, Abdel Rahman Ismail, & Shafik Nada, 2012; Hoque & Hoque, 2011; Kavosi et al., 2014; Kinyoki et al., 2015; Kumar et al., 2008; Scholl, 2005; Singh & Patra, 2014; Zangmo, de Onis, & Dorji, 2012). Food insecurity adversely affects individuals in both resource‐poor and resource‐rich environments (FAO, 2010). Though food insecurity can affect any one, its effect on women deserves special attention because of their social vulnerability to it.

Though a good understanding of the factors that contribute to household food insecurity (HFI) is critical for designing effective strategies to address poor maternal dietary intake, there is limited information on the magnitude and determinants of HFI and how it relates to the nutritional status of pregnant women in Northern Ghana. This study therefore assessed household food insecurity, its determinants and relationship with nutritional status of pregnant women belonging to different gestational ages.

## 2 METHODS

### 2.1 Study area

The study covered 25 communities located in five districts of Northern Ghana. The International Institute of Tropical Agriculture is currently operating in these districts, namely Nadowli, Wa West in the Upper West Region (UWR), Tolon and Savelugu in the Northern Region (NR), and Kassena‐Nankana in the Upper East Region (UER).

The study area is characterized by high poverty and recurrent droughts and floods which predispose communities to increased vulnerability to food insecurity and malnutrition. The Ghana Living Standards Survey Round 6 Report showed that the three study regions have high proportions of households in the lowest quintile than in the highest quintile. The UWR has the highest proportion of households in the lowest quintile (55.7%) and the NR recorded the lowest proportion in the highest quintile (10.2%; GSS, 2014). These are indication that poverty is more prevalent in the three NR, particularly in the UWR.

The three regions share some boundaries with each other. The UER shares its northern boundary with Burkina Faso and its eastern boundary with the Republic of Togo. The UWR on the other hand has a northern boundary with Burkina Faso and a western boundary with La Cote d'Ivoire**.** The UER and UWR share their southern boundaries with the NR which also has la Cote d'Ivoire to the west and Togo to the east.

Majority of the people have agriculture as their main occupation while some are involved in trading. The main staple foods including maize, sorghum, millet and yam are usually harvested from October through December. Although the food security situation is usually good during the harvesting time, child care tends to suffer because of lack of time on the part of rural mothers. A high proportion of rural women work daily away from home, and therefore frequently face challenges to the care of children.

The rainfall pattern is unimodal and the period is usually short and lasts from May to August, followed by a long dry season (September–April) with dry harmattan winds.

### 2.2 Study design, study population, and sampling

This study was an analytical cross sectional survey involving pregnant women in different stages of gestation. The basic primary sampling unit was the household in which there was a pregnant woman. In each community, a complete list of all households was compiled and were serially numbered. Systematic random sampling was then used in selecting study households. To get the sampling interval, the total number of households in a cluster was divided by the cluster size. The first household was randomly selected by picking any number within the sample interval. Subsequent selections were made by adding the sampling interval to the selected number in order to locate the next household to visit. If the selected household does not have a target respondent, then next household was selected using the systematic sampling procedure.

The sample size of 400 was determined on the assumption that 50% of the pregnant mothers experienced food insecurity with 5% marginal error and 95% confidence level and a none response rate of 5%. Based on this assumption, the actual sample size for the study was determined using the formula for one‐point sample estimation:

$$n\;=\;\frac{t^{2}\;\times \;p\left(1\;-\;p\right)}{m^{2}},$$

where *n* = required sample size, *t* = confidence level at 95% (standard value of 1.96), *p* = estimated prevalence of food insecurity in the domain area (50.0%) and *m* = margin of error at 5% (standard value of .05).

### 2.3 Data collection

The data were collected using predesigned and pretested semi‐structured questionnaire. Targeted eligible women were interviewed in the local language in their homes. Data collected included socio‐demographic information including marital status, maternal occupation, nutritional status, and household food security. Mid upper arm circumference (MUAC) tape was used to measure arm circumference and Seca electronic adult scale for maternal weight. Information on gestational age was retrieved from Maternal Health Record Books antenatal care (ANC cards).

In order to ensure reliability and validity of data collected, all field assistants with a minimum qualification of Senior High School were given training for 3 days. The content of the training included objectives and methodology, standard measurement procedures, data recording, recruitment, administration of questionnaires and supervision.

### 2.4 Assessment of household food insecurity

The HFI was quantified using the HFI access scale (HFIAS). The HFIAS was developed for use in developing country settings, and it is quantified by asking respondents about three domains of food insecurity: (1) experiencing anxiety and uncertainty about the household food supply; (2) altering quality of the diet and (3) reducing quantity of food consumed (Ozaltin, Hill, & Subramanian, 2010). In arriving at the domains of food access, nine occurrence questions were asked about changes households made in their diet or food consumption patterns due to limited resources to acquire food in the preceding 30 days.

A household was classified as “food secure” if the responses was “never” to all of the nine items; as “food insecure” if the response were “sometimes” or “always” to one or more of the nine occurrence questions. Based on the responses given to the nine questions and frequency of occurrence over the past 30 days, households were assigned a score that ranges from 0 to 27. A higher HFIAS score is indicative of poorer access to food and greater household food insecurity. Food insecure households were further classified into two groups based on overall distribution of the HFIAS in the sample. The lower the score, the most food secured a household was. HFIAS allows assessment of food poverty (i.e., the inability to obtain healthy affordable food).

Though data was collected based on the HFIAS, the household hunger scale (HHS) was used in most of the analyses because of easy comparison of results across different cultures and greater reliability of responses from respondents. The HHS comprises three subset questions from the HFIAS that pertain to insufficient food quantities (Deitchler et al., 2011; Silventoinen, 2003). Scores of 0–1 are classified as “little to no household hunger”; 2–3 as “moderate household hunger” and 4–6 “severe household hunger” (Silventoinen, 2003). Women with scores 2–6 are therefore classified as experiencing “moderate or severe household hunger.” For logistic regression analyses, the three classes were regrouped into two (none/mild and moderate/severe household food insecurity).

### 2.5 Coping strategies index

The reduced coping strategy index (CSI) is considered a proxy indicator of the food access component of food security and it is calculated on the basis of a specific set of behaviors each with its own universal severity weighting (Maxwell & Caldwell, 2008; Maxwell et al., 2003). The five standard coping strategies and their severity weightings are:

1. eating less preferred/expensive foods (1.0);
2. borrowing food or relying on help from friends and relatives (2.0);
3. limiting portion sizes at meal times (1.0);
4. limiting adult intake so that small children can eat (3.0) and
5. reducing the number of meals per day (1.0).

Answers to the simple question “In the past 7 days, if there have been times when you did not have enough food or enough money to buy food, how many days has your household had to adopt a particular food‐based coping strategy” were used to create the CSI.

For each household, a score was given to each coping strategy. The score = (frequency with which coping strategy is used) × (severity weight). The scores for each coping strategy are added together to give a composite score for each household. Higher values of the index indicate more severe food insecurity.

### 2.6 Nutritional status assessment

Mid upper arm circumference was used to assess the nutritional status of the pregnant women. MUAC was used as a proxy for body weight, since it is not affected by gestational age (Krasovec & Anderson, 1991). MUAC was measured to the nearest 0.1 cm, and values below 25.0 cm were classified in the analyses as an indicator of low body weight.

### 2.7 Determination of household economic status

A household wealth index based on household assets and housing quality was used as a proxy indicator for socio‐economic status (SES) of households. Principal component analysis was used to determine household wealth index from information collected on housing quality (floor, walls, and roof material), source of drinking water, type of toilet facility, the presence of electricity, type of cooking fuel, and ownership of modern household durable goods and livestock (e.g., bicycle, television, radio, motorcycle, sewing machine, telephone, cars, refrigerator, mattress, bed, computer, and mobile phone; Filmer & Pritchett, 2001; Howe, Hargreaves, & Huttly, 2008; Rutstein & Johnson, 2004; Vyas & Kumaranayake, 2006).

These facilities or durable goods are often regarded as modern goods that have been shown to reflect household wealth. A household of zero index score for example means that household had not a single modern good. The scores were thus added up to give the proxy household wealth index.

The main aim of creating the index was to categorize households into SES groupings in order that we could compare the difference in the prevalence of HFI between the groups of lowest and highest SES.

### 2.8 Data analysis

The data were coded for statistical analysis using SPSS for windows 21.0 (SPSS Inc, Chicago). For continuous outcomes, statistical significance was assessed using analysis of variance. For categorical and dichotomous outcomes, chi square tests were performed to assess statistical significance. Multiple regression analysis was used to identify the independent contributors to food insecurity and maternal nutritional status, controlling for potential confounding factors. A *p* value of <.05 was considered statistically significant.

### 2.9 Ethical considerations

Ethical clearance was obtained from the Institutional Review Board of the Tamale Teaching Hospital (ref no. TTH/10/11/15/01). Participation in the study was voluntary and no incentives were provided. Verbal informed consent was sought from all the study participants before the commencement of any interview. The study was not harmful to any study participant. Study participants were free to withdraw from the study at any time without any penalty.

## 3 RESULTS

### 3.1 Socio‐demographic characteristics of the sample

A total of 400 pregnant women were approached and all of them consented and accepted to participate in the study, thus giving a response rate of 100%. The mean age of mothers was 26.2 ± 6.4 years which range from 15 to 48 years. Majority (54.0%) of the respondents were Muslims. Majority, (85.5%) of the respondents were married and (51.8%) of the mothers had no formal education at all. Farming and petty trading were common among the mothers (Table 1).

**Table 1:** Sample characteristics (*N* = 400)

Variable	Frequency (*n*)	Percentage
Age groupings (years)		
15–24	176	46.5
25–34	180	44.6
35–49	44	8.8
Religion		
Christian	176	44.0
Muslim	216	54.0
Traditionalist	8	2.0
Classification of occupation		
Trader	98	24.5
Farmer	123	30.8
Civil servant	2	0.5
Service worker	67	16.8
Education/teacher	5	1.3
Health worker	3	0.8
Nothing	102	25.5
Ethnicity		
Dagomba	119	29.8
Kassena	71	17.8
Dagaba	110	27.5
Kukomba	2	0.5
Frafra	14	3.5
Wala	55	13.8
Fulani	2	0.5
Dorimor	27	6.8
Education level of mother		
None	207	51.8
Low (primary & junior high)	157	39.3
High (≥senior high)	36	9.0
Marital status		
Single	8	2.0
Married	342	85.5
Widowed	5	1.3
Co‐habiting	33	8.3
Separated	12	3.0

### 3.2 Magnitude of HFI and maternal malnutrition

Table 2 shows summary results of the levels of perceived household food insecurity. The majority (77.5%) of households experienced some degree of food insecurity in the month prior to the survey. The prevalence of moderate and severe household hunger was 25.9% (95% CI: 19.0, 34.3) and 6.8% (95% CI: 4.2, 10.9) respectively. Generally, nearly 60% of all households reported worrying about not having enough food in the household, and over 60.0% reported having to eat a limited variety of foods due to lack of resources to buy food in the past 30 days.

**Table 2:** Proportion of households that experienced specific food –insecurity ‐related conditions in the last 30 days preceding the survey (*N* = 400)

	Frequency (*n*)	Proportion (%)
Level of household hunger		
Little to no hunger	288	67.3
Moderate hunger	91	25.9
Severe hunger	21	6.8
Food –insecurity experience		
Worrying that household would not have enough food	225	58.9
Household member not able to eat the kinds of foods preferred because of lack of resources	222	59.9
Household member has to eat a limited variety of foods due to lack of resources	230	62.8
Household member has to eat some food that he/she really did not want to eat because of lack of resources to obtain other types of food	212	57.8
Household member has to eat a smaller meal than is needed because there was not enough food	209	56.5
Household member has to eat fewer meals in a day because there was not enough food	200	55.2
No food to eat of any kind in your household because of lack of resources to get food	141	38.5
Household member go to sleep at night hungry because there was not enough food	121	35.8
Household member go a whole day and night without eating anything because there was not enough food	69	18.4

Based on MUAC cut offs, the overall prevalence of underweight (MUAC <25.0 cm) was 28.8% (95% CI: 24.6, 33.4), while 71.2% of the respondents were classified as normal (MUAC ≥25.0 cm).

### 3.3 Coping strategies adopted to minimize food insecurity

The prevalence of adopting coping strategy to minimize food insecurity at least 1 day in a week is shown in Table 3. Borrowing money to buy food and receiving foods from family members, relatives and friends was the least adopted coping strategy. Limiting portion sizes at meal times at least 1 day in a week was commonly practiced.

**Table 3:** Coping strategies adopted to minimize food insecurity

Coping strategy	Frequency (*n*)	Percentage
Eating less preferred foods		
Never	166	41.5
Once a week	95	23.8
4–6 days/week	100	25.0
Daily	39	9.8
Borrowing food or relying on help from friends and relatives		
Never	283	71.6
Once/week	59	14.9
4–6 days/week	44	11.1
Daily	9	2.3
Limiting portion sizes at meal times		
Never	201	50.5
Once/week	107	26.9
4–6 days/week	73	18.3
Daily	17	4.3
Limiting adult intake so that small children can eat		
Never	214	53.9
Once/week	83	20.9
4–6 days/week	85	21.4
Daily	15	3.8
Reducing the number of meals per day		
Never	214	54.3
Once/week	95	24.1
4–6 days/week	73	18.5
Daily	12	3.0

### 3.4 Demographic, socioeconomic, and coping strategy variables associated with household food insecurity

Correlation analysis showed that CSI was positively associated with overall HFI as measured by HHS among pregnant women in rural households (*r* = .60, *p* < .001). However, CSI associated negatively with dietary diversity score for women (*r* = −.17, *p* < .001). HHS score also associated negatively with dietary diversity score for women (*r* = −.14, *p* = .004).

In Pearson correlation analyses, the CSI was negatively associated with consumption of staples, dairy, flesh foods, vitamin A rich dark green leafy vegetables and other vegetables. Table 4 shows the correlation of CSI and the consumption of selected foods groups.

**Table 4:** Correlation between coping strategy index and the consumption of selected foods

	Vitamin A rich dark vegetables	Consumption scores of foods							
		Staple food	Beans	Seeds and nuts	Dairy	Flesh foods	Egg	Other vegetables	Other fruits
Correlation	−.11*	−.13**	−.05	.08	−.12*	−.14**	−.03	−.14**	−.04
Sig. (two‐tailed)	.03	.007	.340	.11	.02	.007	.55	.004	.42
*N*	400	400	398	400	399	400	399	400	400

*Correlation is significant at the .05 level (two‐tailed). **Correlation is significant at the .01 level (two‐tailed).

Similarly, analysis of the type of food consumed by the pregnant women in the past 24 hr prior to the study revealed that participants who were food insecure had less frequent intakes of vegetables compared with food‐secure participants *F*(1, 398) = 10.2, *p* = .002. There was a strong and significant negative association between the consumption of animal products (e.g., meat, chicken, and fish) in the past 24 hr and food insecurity status *F*(1, 398) = 15.4, *p* < .001. There was no discernible difference in the consumption of egg, fruit and legume across the food insecurity groups.

Household food insecurity was significantly higher in Kasena‐Nankana/Bongo, compared to Nadowli District (56.0% vs. 14.0%) (Table 5). Compared to women from food‐secure households, women from households experiencing food insecurity were more likely to be poor as indicated by low household wealth index chi‐square (χ^2^) = 41.0, *p* < .001. Households with greater numbers of children under 5 years of age were more likely of being food insecure, compared with households with fewer under‐fives (χ^2^ = 13.8, *p* = .003).

**Table 5:** Bivariate analysis of variables associated with household food insecurity

Variable	*N*	Classification of household hunger scale		Test statistic
		Little to no household hunger *n* (%)	Moderate to severe household hunger *n* (%)	
District				
Tolon	76	48 (63.2)	28 (36.8)	χ^2^ = 54.8, *p* < .001
Savelugu	50	43 (86.0)	7 (14.0)	
Kasena‐Nankana/Bongo	84	37 (44.0)	47 (56.0)	
Wa West	107	92 (86.0)	15 (14.0)	
Nadowli	83	68 (81.9)	15 (18.1)	
No. of under‐five children in household				
None	100	83 (83.0)	17 (17.0)	χ^2^ = 13.8, *p* = .003
1–2	203	144 (70.9)	59 (29.1)	
3–4	72	49 (68.1)	23 (31.9)	
>4	25	12 (48.0)	13 (52.0)	
Type of occupation				
None	**102**	62 (60.8)	40 (39.2)	Fisher's exact test = 8.2, *p* = .013
Non‐government	**288**	218 (75.7)	70 (24.3)	
Government	**10**	8 (80.0)	2 (20.0)	
Household wealth index				
Low	223	132 (59.2)	91 (40.8)	χ^2^ = 41.0, *p* < .001
High	177	156 (88.1)	21 (11.9)	
Timing of first ANC visit				
First trimester	272	188 (68.1)	84 (30.9)	Fisher's exact test = 9.7, *p* = .007
Second trimester	118	96 (81.4)	22 (18.6)	
Third trimester	9	4 (44.4)	5 (55.6)	
Marital status				
Unmarried	58	36 (62.1)	22 (37.9)	χ^2^ = 3.3, *p* = .08
Married	342	252 (73.7)	90 (26.3)	
Eating less preferred foods				
No	166	151 (91.0)	15 (9.0)	χ^2^ = 50.6, *p* < .001
Yes	234	137 (58.5)	97 (41.5)	
Borrowing food or relying on help from friends and relatives				
No	283	241 (85.2)	42 (14.8)	χ^2^ = 81.3, *p* < .001
Yes	112	45 (40.2)	67 (59.8)	
Limiting portion sizes at meal times				
No	201	182 (90.5)	19 (9.5)	χ^2^ = 68.6, *p* < .001
Yes	197	105 (53.3)	92 (46.7)	
Limiting adult intake so that small children can eat				
No	214	190 (88.8)	24 (11.2)	χ^2^ = 66.2, *p* < .001
Yes	183	95 (51.9)	88 (48.1)	
Reducing the number of meals per day				
No	214	204 (95.3)	10 (4.7)	χ^2^ = 123.7, *p* < .001
Yes	180	81 (45.0)	99 (55.0)	

Coping strategies adopted by the households to minimize the impact of food insecurity included borrowing food or relying on help from friends and relatives, relying on less expensive foods, limiting portion size of meals and reducing numbers of meals eaten in a day.

Logistic regression analysis showed that households with larger number of children (>4) under 5 years were six times more likely (AOR = 6.85, CI: 2.13, 22.05) to be food insecure (Table 6).

**Table 6:** Determinants of household food insecurity

	Wald	Sig.	Exp(β)	95% CI for exp(β)
				(lower, upper)
Low household wealth index	30.577	<.001	5.14	(2.88, 9.18)
Under‐5 in household (reference: none)	11.627	.009		
1‐2	6.314	.012	2.47	(1.22, 5.005)
3‐4	5.814	.016	3.12	(1.24, 7.87)
>4	10.390	.001	6.85	(2.13, 22.05)
District of residence (reference: Nadowli)	46.167	<.001		
Tolon	1.250	.26	1.62	(0.70, 3.74)
Savelugu	2.604	.11	0.41	(0.14, 1.21)
Kasena‐Nankana/Bongo	23.431	<.001	6.91	(3.16, 15.11)
Wa West	0.026	.87	0.93	(0.41, 2.14)
Constant	47.782	<.001	0.036	

The set of variables accounted for 33.3% of the variance in household food insecurity (Nagelkerke *R* ^2^ = .33).

Compared to the Nadowli District, women resident in the Kasena‐Nankana/Bongo District were six times more likely of experiencing food insecurity (AOR = 6.91, CI: 3.16, 15.11) and households having lower wealth index (AOR = 5.14, CI: 2.88, 9.18) were five times more likely to be food insecure.

### 3.5 Determinants of maternal nutritional status

Bivariate analyses showed that gestational age of woman, type of residence, occupation of mother, and age of mother were associated with maternal thinness (Table 7).

**Table 7:** Bivariate analyses factors associated with maternal thinness during pregnancy

Characteristic	*N*	Classification of MUAC		Test statistic
		Underweight (<25 cm) *n* (%)	Normal (at least 25 cm) *n* (%)	
District of residence				
Tolon	76	25 (32.9)	51 (67.1)	χ^2^ = 12.5, *p* < .01
Savelugu	50	14 (28.0)	36 (72.0)	
Kasena‐Nankana/Bongo	84	28 (33.3)	56 (66.7)	
Wa West	107	16 (15.0)	91 (85.0)	
Nadowli	83	17 (20.5)	66 (79.5)	
Gestational age of woman				
First trimester	63	10 (15.9)	53 (84.1)	χ^2^ = 9.0, *p* = .01
Second trimester	143	29 (20.3)	114 (79.7)	
Third trimester	193	61 (31.6)	132 (68.4)	
Type of occupation				
None	102	38 (37.3)	64 (62.7)	Fisher's exact test = 10.4, *p* = .004
Non‐government	288	60 (20.8)	228 (79.2)	
Government	10	2 (20.0)	8 (80.0)	
Age of mother (years)				
Under 20	53	25 (47.2)	28 (52.8)	Fisher's exact test = 20.9, *p* < .001
20–35	313	73 (23.3)	240 (76.7)	
35+	34	2 (5.9)	32 (94.1)	

MUAC, Mid upper arm circumference.

Though household wealth index significantly associated with food security, it did not associate with maternal nutritional status during pregnancy. Maternal educational level was also not associated with maternal thinness. The risk of maternal thinness increased as the gestational age increased. Both dietary diversity and household food security were not significantly associated with maternal nutritional status during pregnancy among this sample of rural women.

The independent predictors of maternal thinness were region of residence, gestational age and maternal age (Table 8). Gestational age was the greatest predictor of maternal thinness. Compared to women in the first trimester, women in the third trimester were 2.2 times more likely of being underweight (AOR = 2.19, CI: 1.02, 4.70). Women who were under 20 years of age were 11.9 times more likely of being thin compared to women aged more than 35 years (AOR = 11.97, CI: 2.55, 5. 67).

**Table 8:** Predictors of maternal thinness (logistic regression analysis)

	Wald	Sig.	Exp(β)	95% CI for exp(β)
				(lower, upper)
Region (reference: upper west)	6.99	.03		
Northern	5.17	.02	1.90	(1.09, 3.29)
Upper east	4.92	.027	2.00	(1.08, 3.68)
Gestation (reference: first trimester)	6.76	.03		
Second trimester	0.28	.60	1.25	(0.55, 2.81)
Third trimester	4.05	.04	2.19	(1.02, 4.70)
Maternal age (reference: >35 years)	14.76	.001		
Under 20	9.89	.002	11.97	(2.55, 5.67)
20–35	4.04	.044	4.51	(1.04, 19.60)
Constant	18.96	.000	0.029	

## 4 DISCUSSION

In this study, we assessed household food insecurity, its determinants and relationship with nutritional status of pregnant women belonging to different gestational ages. The relationship among coping strategy, HFI and dietary diversity was also investigated.

### 4.1 Magnitude and determinants of household food insecurity

The prevalence of perceived moderate to severe household hunger was quite high. Generally, nearly 60% of all households reported worrying about not having enough food in the household, and over 60.0% reported having to eat a limited variety of foods due to lack of resources to buy food in the past 30 days prior to the study.

The determinants associated with increased risk of high food insecurity were district of residence, households of lower wealth index, and household size (more than four children in the household).

Respondents of low household wealth tercile were significantly more likely to experience food insecurity. This finding is consistent with other findings (Arimond et al., 2011; Mascie‐Taylor et al., 2010). We also found a positive association between household wealth index and dietary diversity score of the pregnant women, supporting findings from a comparative study from Kenya, the Philippines, and Bangladesh (Bouis, Eozenou, & Rahman, 2011).

Compared to the Nadowli District, women resident in the Kasena‐Nankana and Bongo Districts were six times more likely of experiencing food insecurity. The UER, where the two districts are located is reported to experience annual transitory food insecurity.

### 4.2 Association among coping strategy, HFI, and dietary diversity

The CSI which is a measure of food insecurity was negatively associated with overall household dietary diversity, a relationship that had been earlier reported in Malawi (Jones, 2015). HFI as measured by the HHS was also associated negatively with dietary diversity score for pregnant women. Compared to food‐secure households, food‐insecure households were less likely to consume flesh foods, dairy products, staples, vitamin A rich dark green leafy vegetables, and other vegetables. This strongly support available evidence that food insecurity is associated with less diverse diets in many regions including Africa, South Asia, and Latin America (Hoddinott & Yohannes, 2002; Jones, 2015).

### 4.3 Relationship between food security and adequate nutrition

Though HFI was negatively associated with overall household dietary diversity, it was unrelated to maternal underweight in this study population.

The multifaceted nature of malnutrition has led some scholars to conclude that though food security is necessary, it is not sufficient to ensure adequate nutrition and to prevent malnutrition (Pinstrup‐Andersen, 2013; Ruel & Alderman, 2013) because of the role of other food and nonfood factors.

Measures to effectively implement homestead food production coupled with nutrition education to increase availability and consumption of micronutrient‐rich foods (fruits and vegetables and animal source foods) in poor households may contribute to the nutritional well‐being of the population.

## 5 CONCLUSIONS

Though perceived food insecurity was highly prevalent, it was not associated with maternal thinness of this sample of rural pregnant women. The risk of maternal thinness increased as the gestational age increased and this has a great potential of adversely influencing pregnancy outcomes and overall quality of life.

## 6 STUDY LIMITATIONS

Information on dietary diversity and CSI was based on the respondents’ recall of events and so recall bias cannot be ruled out. Nevertheless, simple indicators of food security have been shown to provide adequate variance for determining nutrition‐related outcomes (Tiwari, Emmanuel, & Maya, 2013). Exact nutrient intakes could not be calculated from the available data because the 24‐hr diet recall used was not quantitative food frequency data.

## References

[fsn3506-bib-0001] Abu‐Saad, K. , & Fraser, D. (2010). Maternal nutrition and birth outcomes. Epidemiologic Reviews, 32(1), 5–25. 20237078 10.1093/epirev/mxq001

[fsn3506-bib-0002] Arimond, M. , Hawkes, C. , Ruel, M. T. , Sifri, Z. , Berti, P. R. , Leroy, J. L. , … Frongillo, E. A. (2011). Agricultural interventions and nutrition: Lessons from the past and new evidence In Thompson B. & Amoroso L. (Eds.), Combating micronutrient deficiencies: Food‐based approaches (pp. 41–75). FAO.

[fsn3506-bib-0003] Black, R. E. , Allen, L. H. , Bhutta, Z. A. , et al. (2008). Maternal and child undernutrition: Global and regional exposures and health consequences. Lancet, 371, 243–260. 18207566 10.1016/S0140-6736(07)61690-0

[fsn3506-bib-0004] Bouis, H. E. , Eozenou, P. , & Rahman, A. (2011). Food prices, household income, and resource allocation: Socioeconomic perspectives on their effects on dietary quality and nutritional status. Food and Nutrition Bulletin, 32(Suppl 1), 14S–23S. 10.1177/15648265110321S103 21717914

[fsn3506-bib-0005] Deitchler, M. , Ballard, T. , & Swindale, A. , et al. (2011). Introducing a simple measure of household hunger for cross‐cultural use. Technical Note No. 12. Washington, DC: Food and Nutrition Technical Assistance II Project (FANTA‐2).

[fsn3506-bib-0006] El‐Farrash, R. A. , Abdel Rahman Ismail, E. , & Shafik Nada, A. (2012). Cord blood iron profile and breast milk micronutrients in maternal iron deficiency anemia. Pediatric Blood & Cancer, 58(2), 233–238. 21548016 10.1002/pbc.23184

[fsn3506-bib-0007] FAO . (2010). The state of food insecurity in the world: Addressing food insecurity in protracted crises. Rome, Italy: FAO.

[fsn3506-bib-0008] Filmer, D. , & Pritchett, L. H. (2001). Estimating wealth effects without expenditure data—or tears: An application to educational enrollments in states of India. Demography, 38, 115–132. 11227840 10.1353/dem.2001.0003

[fsn3506-bib-0009] GSS . (2014). Ghana living standards survey. Round 6. Accra, Ghana: Ghana Statistical Service (GSS).

[fsn3506-bib-0010] Hoddinott, J. , & Yohannes, Y. (2002). Dietary diversity as a food security indicator. Washington, DC: International Food Policy Research Institute.

[fsn3506-bib-0011] Hoque, M. , & Hoque, M. E. (2011). Knowledge of danger signs for major obstetric complications among pregnant KwaZulu‐Natal women: Implications for health education. Asia‐Pacific Journal of Public Health, 23(6), 946–956. 22144713 10.1177/1010539511428698

[fsn3506-bib-0012] Howe, L. D. , Hargreaves, J. R. , & Huttly, S. R. A. (2008). Issues in the construction of wealth indices for the measurement of socio‐economic position in low‐income countries. Emerging Themes in Epidemiology, 5, 3. 18234082 10.1186/1742-7622-5-3 PMC2248177

[fsn3506-bib-0013] Jones, A. D. (2015). Household food insecurity is associated with heterogeneous patterns of diet quality across urban and rural regions of Malawi. World Medical & Health Policy, 7(3), 234–254.

[fsn3506-bib-0014] Kavosi, E. , Hassanzadeh Rostami, Z. , Kavosi, Z. , et al. (2014). Prevalence and determinants of under‐nutrition among children under six: A cross‐sectional survey in Fars province, Iran. International Journal of Health Policy and Management, 3(2), 71–76. 25114945 10.15171/ijhpm.2014.63 PMC4122078

[fsn3506-bib-0015] Kinyoki, D. K. , Berkley, J. A. , Moloney, G. M. , et al. (2015). Predictors of the risk of malnutrition among children under the age of 5 years in Somalia. Public Health Nutrition, 19, 1–9. 10.1017/S1368980015001913 PMC4697134 26091444

[fsn3506-bib-0016] Krasovec, K. , & Anderson, M. (Eds.). (1991). Maternal nutrition and pregnancy outcomes: Anthropometric assessment. Washington, DC: Pan American Health Organization (PAHO).

[fsn3506-bib-0017] Kumar, A. , Rai, A. K. , Basu, S. , et al. (2008). Cord blood and breast milk iron status in maternal anemia. Pediatrics, 121(3), e673–e677. 18310187 10.1542/peds.2007-1986

[fsn3506-bib-0018] Mascie‐Taylor, C. , Marks, M. , Goto, R. , et al. (2010). Impact of a cash‐for‐work programme on food consumption and nutrition among women and children facing food insecurity in rural Bangladesh. Bulletin of the World Health Organization, 88(11), 854–860. 21076567 10.2471/BLT.10.080994 PMC2971521

[fsn3506-bib-0019] Maxwell, D. , & Caldwell, R. (2008). The Coping Strategies Index: Field methods manual (2nd ed.). USA: Cooperative for Assistance and Relief Everywhere Inc.

[fsn3506-bib-0020] Maxwell, D. , Watkins, B. , Wheeler, R. , et al. (2003). The Coping Strategies Index: Field methods manual. Rome, Italy: CARE, WFP.

[fsn3506-bib-0021] Ozaltin, E. , Hill, K. , & Subramanian, S. V. (2010). Association of maternal stature with offspring mortality, underweight, and stunting in low‐ to middle‐income countries. Journal of the American Medical Association, 303(15), 1507. 20407060 10.1001/jama.2010.450 PMC3100588

[fsn3506-bib-0022] Pinstrup‐Andersen, P. (2013). Can agriculture meet future nutrition challenges? The European Journal of Development Research, 25, 5–12.

[fsn3506-bib-0023] Ruel, M. T. , & Alderman, H. (2013). Nutrition‐sensitive interventions and programmes: How can they help to accelerate progress in improving maternal and child nutrition? Lancet, 382, 536. 23746780 10.1016/S0140-6736(13)60843-0

[fsn3506-bib-0024] Rutstein, S. O. , & Johnson, K. (2004). DHS comparative reports 6: The DHS Wealth Index. Calverton, MD: ORC Macro, MEASURE DHS.

[fsn3506-bib-0025] Scholl, T. O. (2005). Iron status during pregnancy: Setting the stage for mother and infant”. The American Journal of Clinical Nutrition, 81(5), 1218S–1222S. 15883455 10.1093/ajcn/81.5.1218

[fsn3506-bib-0026] Silventoinen, K. (2003). Determinants of variation in adult body height. Journal of Biosocial Science, 35(2), 263–285. 12664962 10.1017/s0021932003002633

[fsn3506-bib-0027] Singh, R. K. , & Patra, S. (2014). Extent of anaemia among preschool children in EAG States, India: A challenge to policy makers. Anemia, 2014, Article ID 868752, 9 pages, 2014. https://doi.org/10.1155/2014/868752. 10.1155/2014/868752 PMC4129919 25140250

[fsn3506-bib-0028] Tiwari, S. , Emmanuel, S. , & Maya, S. (2013). Shorter, cheaper, quicker, better: Linking measures of household food security to nutritional outcomes in Bangladesh, Nepal, Pakistan, Uganda, and Tanzania. Washington, DC: World Bank.

[fsn3506-bib-0029] Vyas, S. , & Kumaranayake, L. (2006). Constructing socio‐economic status indices: How to use principal components analysis. Health Policy and Planning, 21, 459–468. 17030551 10.1093/heapol/czl029

[fsn3506-bib-0030] Zangmo, U. , de Onis, M. , & Dorji, T. (2012). The nutritional status of children in Bhutan: Results from the 2008 National Nutrition Survey and trends over time. BMC Pediatrics, 12, 151. 22992335 10.1186/1471-2431-12-151 PMC3507860

